# Pituitary Adenylate Cyclase-Activating Polypeptide Modulates Hippocampal Synaptic Transmission and Plasticity: New Therapeutic Suggestions for Fragile X Syndrome

**DOI:** 10.3389/fncel.2019.00524

**Published:** 2019-11-27

**Authors:** Lucia Ciranna, Lara Costa

**Affiliations:** ^1^Department of Biomedical and Biotechnological Sciences, University of Catania, Catania, Italy; ^2^Department of Clinical and Experimental Medicine, University of Messina, Messina, Italy

**Keywords:** pituitary adenylate cyclase-activating polypeptide, fragile X syndrome, cyclic adenosine monophosphate, long-term depression induced by metabotropic glutamate receptor, hippocampus, learning

## Abstract

Pituitary adenylate cyclase-activating polypeptide (PACAP) modulates glutamatergic synaptic transmission and plasticity in the hippocampus, a brain area with a key role in learning and memory. In agreement, several studies have demonstrated that PACAP modulates learning in physiological conditions. Recent publications show reduced PACAP levels and/or alterations in PACAP receptor expression in different conditions associated with cognitive disability. It is noteworthy that PACAP administration rescued impaired synaptic plasticity and learning in animal models of aging, Alzheimer’s disease, Parkinson’s disease, and Huntington’s chorea. In this context, results from our laboratory demonstrate that PACAP rescued metabotropic glutamate receptor-mediated synaptic plasticity in the hippocampus of a mouse model of fragile X syndrome (FXS), a genetic form of intellectual disability. PACAP is actively transported through the blood–brain barrier and reaches the brain following intranasal or intravenous administration. Besides, new studies have identified synthetic PACAP analog peptides with improved selectivity and pharmacokinetic properties with respect to the native peptide. Our review supports the shared idea that pharmacological activation of PACAP receptors might be beneficial for brain pathologies with cognitive disability. In addition, we suggest that the effects of PACAP treatment might be further studied as a possible therapy in FXS.

## Introduction

Pituitary adenylate cyclase-activating polypeptide (PACAP) was initially discovered in ovine hypothalamus as an endocrine regulator (Miyata et al., 1989). PACAP is highly expressed in the brain and in peripheral tissues in two forms of 38 and 27 amino acid residues, PACAP-38 and PACAP-27 (Arimura et al., 1991; Koves et al., 1991). In the present review, “PACAP” refers to PACAP-38, the most abundant form in the brain (Arimura et al., 1991; Vaudry et al., 2009). We will specifically indicate PACAP-38 and PACAP-27 to underline differences between the two forms.

Pituitary adenylate cyclase-activating polypeptide activates two main classes of G protein-coupled receptors: PAC1 and VPAC (Vaudry et al., 2009). PAC1 is PACAP specific, with a high nanomolar affinity for PACAP and a 1,000-fold lower affinity for the structurally related vasoactive intestinal peptide (VIP). VPAC receptors include VPAC1 and VPAC2 subtypes, with equally high nanomolar affinity for PACAP and VIP. PAC1 and VPAC receptors are expressed in peripheral tissues and in the central nervous system (CNS), where PAC1 is the most abundant (Jolivel et al., 2009). All PACAP/VIP receptors are positively coupled to adenylate cyclase; PAC1 receptors can also activate phospholipase C and Ca^2+^ release. The brain localization, pharmacological features, signal transduction mechanisms, and biological effects of PACAP/VIP receptors are described in details in excellent reviews (Dickson and Finlayson, 2009; Vaudry et al., 2009; Harmar et al., 2012; Hirabayashi et al., 2018).

In the CNS, PACAP is a neurotrophic and a neuroprotective factor regulating differentiation of neuronal precursors, promoting neuronal survival, and exerting neuroprotective effects after brain damage (Arimura et al., 1994; Shioda and Nakamachi, 2015; Reglodi et al., 2018c). Acting on the brain, PACAP also regulates important physiological functions, among which are feeding (Sekar et al., 2017), circadian rhythm (Holland et al., 2018), body temperature (Tan et al., 2016), learning, and memory (see below). We will initially describe the physiological role of PACAP on learning and then highlight recent findings showing an involvement of PACAP in cognitive disability. Finally, we will discuss the possibility to use PACAP as a pharmacological tool in conditions of learning impairment.

## Pituitary Adenylate Cyclase-Activating Polypeptide Modulates Hippocampal Synaptic Transmission, Synaptic Plasticity, and Learning in Physiological Conditions

Pituitary adenylate cyclase-activating polypeptide receptors are highly expressed in rat hippocampus (Shioda et al., 1997; Jaworski and Proctor, 2000; Joo et al., 2004). On cultured hippocampal neurons, PACAP stimulated axon outgrowth (Ogata et al., 2015) and increased the size and density of dendritic spines (Hayata-Takano et al., 2019). Accordingly, PACAP-deficient mice displayed a reduced hippocampal spine density with respect to wild type (Hayata-Takano et al., 2019), indicating an important role of PACAP on synapse formation.

Pituitary adenylate cyclase-activating polypeptide increases the firing rate of hippocampal neurons (Di Mauro et al., 2003; Liu et al., 2003) and inhibits potassium currents responsible for membrane repolarization (Taylor et al., 2014; Gupte et al., 2015), which likely accounts for PACAP-induced increase in intrinsic excitability. Importantly, PACAP modulates hippocampal synaptic transmission and plasticity (Yang et al., 2010) and hippocampus-dependent learning (Borbely et al., 2013). In CA1 neurons, PACAP dose dependently modulates glutamate-mediated transmission (Kondo et al., 1997; Roberto et al., 2001; Ciranna and Cavallaro, 2003; Ster et al., 2009), exerting different effects on AMPA (Costa et al., 2009; Toda and Huganir, 2015) and NMDA receptor-mediated synaptic responses (Yaka et al., 2003; Macdonald et al., 2005). PACAP also stimulates acetylcholine release in the rodent hippocampus (Masuo et al., 1993), which in turn affects glutamate-mediated transmission (Roberto and Brunelli, 2000; Roberto et al., 2001; Pecoraro et al., 2017).

Pituitary adenylate cyclase-activating polypeptide-deficient mice show reduced long-term depression (LTP) in the dentate gyrus (Matsuyama et al., 2003) and impaired memory (Ago et al., 2013; Takuma et al., 2014). Impaired hippocampal LTP was also observed in PAC1 receptor-deficient mice (Otto et al., 2001; Matsuyama et al., 2003), together with a specific deficit in contextual fear conditioning, an index of associative memory, but not in Morris water maze test performance, indicative of spatial discrimination (Sauvage et al., 2000; Otto et al., 2001). In wild-type rats, intravenous injection of PACAP improved spatial memory (Ladjimi et al., 2019); direct infusion of PACAP in the hippocampus and amygdala improved learning in contextual fear conditioning (Schmidt et al., 2015); intracerebroventricular administration of PACAP exerted bi-directional effects (initial impairment and later improvement) on fear conditioning memory (Meloni et al., 2016, 2018) and, at low doses, improved learning in passive avoidance response test (Sacchetti et al., 2001).

## Dysregulation of Pituitary Adenylate Cyclase-Activating Polypeptide Functions in Conditions of Cognitive Impairment

Recent studies show reduced brain levels of PACAP and/or altered PACAP receptor expression in cognitive deficit (Table 1). PACAP-KO mice display neuronal apoptosis, oxidative damage, and neuroinflammation similar to those observed at old age; thus, PACAP deficiency has been proposed as a model of premature aging (Reglodi et al., 2018b). Accordingly, age-related cognition impairment was associated with reduced levels of PACAP in rhesus macaque brain (Han et al., 2017) and in an invertebrate model of aging, in which memory loss was rescued by activation of PAC1 receptors (Pirger et al., 2014).

**TABLE 1:** Involvement of PACAP in conditions involving cognitive impairment.

**Condition**	**Data from human patients**	**Data from animal models**	**References**
Aging	–	Aged pond snail (*Lymnaea stagnalis*): low brain levels of PACAP. PACAP administration rescued learning impairment.	Pirger et al., 2014
		PACAP-KO mice: neuronal apoptosis, oxidative damage, and neuroinflammation.	Reglodi et al., 2018b
		Aged rhesus macaque: reduced brain levels of PACAP (striatum, parietal, and temporal lobes).	Han et al., 2017
Alzheimer’s disease (AD)		Transgenic AD mouse models: reduced PACAP gene expression.	Wu et al., 2006
		Transgenic AD mouse model: *in vivo* administration of PACAP exerted neuroprotective effects and improved learning.	Rat et al., 2011
	AD patients: reduced brain levels of PACAP.		Han et al., 2014a, b, 2015
		Transgenic AD mouse model: reduced PACAP gene expression and brain levels. PACAP protected cultured neurons against Aβ toxicity.	Han et al., 2014b, 2017
Parkinson’s disease (PD)	–	6-OHDA-treated rats: *in vivo* administration of PACAP exerted neuroprotective effects and reduced behavioral deficits.	Reglodi et al., 2004, 2006
		MPTP-treated mice: *in vivo* administration of PACAP exerted neuroprotective effects.	Wang et al., 2008; Lamine et al., 2016; Lamine-Ajili et al., 2016
		MPTP-treated mice: *in vivo* administration of PACAP improved learning.	Deguil et al., 2010
		Prostaglandin J2-treated mice: PACAP-27 exerted neuroprotective effects.	Shivers et al., 2014
		MPTP-treated macaque: altered PAC1 receptor expression in basal ganglia	Feher et al., 2018
Huntington’s disease (HD)	HD patients: reduced PAC1 receptor expression in the hippocampus.	Transgenic HD mouse models: reduced expression of PAC1, VPAC1, and VPAC2 receptors in the hippocampus. *In vivo* administration of PACAP rescued synapse formation, PAC1 receptor levels, and learning.	Cabezas-Llobet et al., 2018
Schizophrenia	Schizophrenia patients: mutations of genes coding for PACAP and PAC1 receptors.	–	Hashimoto et al., 2007
Fragile X syndrome	–	*Fmr1* KO mouse hippocampal slices: PACAP rescued abnormal synaptic plasticity.	Costa et al., 2018

*PACAP, pituitary adenylate cyclase-activating polypeptide; AD, Alzheimer’s disease; 6-OHDA, 6-hydroxydopamine; MPTP, 1-methyl-4-phenyl-1,2,3,6-tetrahydropyridine.*

Defects in PACAP-mediated functions are well documented in Alzheimer’s disease (AD), the most devastating neurodegenerative disease leading to memory loss and dementia. Reduced PACAP levels were observed in the brain of different transgenic AD mouse models (Wu et al., 2006; Han et al., 2014b, 2017) and in cortical brain samples from AD patients, where PACAP levels were inversely related to the amount of amyloid plaques and neurofibrillary tangle, as well as to dementia rating scores (Han et al., 2014a). Reduced PACAP levels and altered PAC1 receptor expression in the brain of AD patients were detected since early stages of the progressive neurodegeneration characterized by mild cognitive impairment (Han et al., 2015). Interestingly, besides exerting a neuroprotective role, PACAP stimulates a non-amyloidogenic processing pathway of amyloid precursor protein (APP) (Kojro et al., 2006), suggesting that PACAP might be used in AD therapy. Administration of PACAP has proven to be effective against Aβ-induced toxicity in different AD mouse models (Rat et al., 2011; Han et al., 2014b). Intranasal administration of PACAP to APP-transgenic mice increased brain expression of PACAP and PACAP receptors, stimulated the production of neurotrophic and antiapoptotic factors [brain-derived neurotrophic factor (BDNF) and Bcl-2], enhanced the expression of the Aβ-degrading enzyme neprilysin, and improved learning (Rat et al., 2011). Accordingly, VIP decreased amyloid plaques and prevented brain atrophy in the 5xFAD mouse model of AD (Korkmaz et al., 2018). The same authors suggest that VIP-mediated neuroprotective effects might also be used for therapy of Parkinson’s disease (PD) (Korkmaz and Tuncel, 2018).

Parkinson’s disease, a neurodegenerative disorder characterized by loss of dopaminergic neurons in the substantia nigra, primarily affects motor control and also involves cognition deficit (Aarsland et al., 2017). A new study shows a decreased PAC1 receptor expression in basal ganglia in a macaque model of PD (Feher et al., 2018), suggesting that reduced PACAP function contributes to neurodegeneration and PACAP might become a promising tool for PD therapy (Reglodi et al., 2017). Administration of PACAP to rats treated with the neurotoxin 6-hydroxydopamine (6-OHDA), a model of PD, prevented degeneration of nigral dopaminergic neurons and rescued behavioral deficits (Reglodi et al., 2004, 2006). Likewise, in a different murine PD model [mice treated with 1-methyl-4-phenyl-1,2,3,6-tetrahydropyridine (MPTP)], intravenous injection of PACAP-27 prevented neuronal loss in the substantia nigra (Wang et al., 2008) and rescued learning deficit (Deguil et al., 2010). Still in MPTP-treated mice, PACAP treatment exerted neuroprotective effects in the substantia nigra (Lamine et al., 2016) and reduced abnormal autophagy, a mechanism that might contribute to neuronal death (Lamine-Ajili et al., 2016). PACAP-27 also prevented dopaminergic neuronal loss and motor deficit in prostaglandin J2-treated mice, another proposed PD model (Shivers et al., 2014).

A reduced expression of all PACAP receptor subtypes was observed in two different mouse models of Huntington’s disease (HD), an inherited degenerating motor and cognitive disease, and PAC1 receptors were downregulated in postmortem hippocampal samples from HD patients (Cabezas-Llobet et al., 2018). Remarkably, intranasal administration of PACAP to HD mice increased PAC1 receptor expression, stimulated BDNF production, reduced the formation of huntingtin aggregates, prevented the loss of hippocampal glutamatergic synapses, and improved memory (Cabezas-Llobet et al., 2018).

Finally, mutations of the genes coding for PACAP and for PAC1 receptors were found in schizophrenic patients together with reduced hippocampal volume and impaired memory (Hashimoto et al., 2007). Schizophrenia involves dysregulation of brain dopaminergic system (Weinstein et al., 2017). Interestingly, genetic ablation of D3 receptors increased the expression of PACAP and PACAP receptors in mouse hippocampus and enhanced memory (Marzagalli et al., 2016), suggesting a close interplay between PACAP and dopamine on learning and memory.

## Pituitary Adenylate Cyclase-Activating Polypeptide Rescues Synaptic Plasticity in a Mouse Model of Fragile X Syndrome

Fragile X syndrome (FXS) is a genetic form of intellectual disability affecting 1/4,000 males and 1/6,000 females. FXS patients show cognitive and language deficits; subgroups of patients also display autistic features, epilepsy, attention deficit and hyperactivity disorder (ADHD), and mood disorders (Bardoni et al., 2006; Maurin et al., 2014; Gross et al., 2015; Hagerman et al., 2017). FXS is caused by transcriptional silencing of the FMR1 gene coding for fragile X mental retardation protein (FMRP) (Verkerk et al., 1991), an mRNA-binding protein mostly functioning as a repressor (Garber et al., 2006) and in some cases as an enhancer (Bechara et al., 2009) of protein translation (Darnell and Klann, 2013). Hundreds of mRNAs have been identified as FMRP targets, particularly mRNAs coding for proteins involved in synapse development and function (Bassell and Warren, 2008; Bear et al., 2008). Cortical neurons from *Fmr1* knock out (*Fmr1* KO) animal models of FXS (Comery et al., 1997) and FXS patients (Irwin et al., 2000) display an increased density of dendritic spines, with a long and thin morphology reminiscent of immature filopodia. Abnormal dendritic spine morphology has crucial consequences on synaptic function. Many alterations of glutamate-mediated synaptic transmission and plasticity were found in the brain of *Fmr1* KO mice. Among the first discovered, hippocampal LTP induced by metabotropic glutamate receptors (mGluR-LTD) is abnormally enhanced (Huber et al., 2002). Exaggerated mGluR-LTD led to formulation of the “mGluR theory” of FXS, pointing out excessive signaling downstream activation of mGluRs (Bear et al., 2004). In *Fmr1* KO neurons, mGluRs also show altered cell-surface mobility, abnormal coupling to NMDA receptors, and impaired mGluR-LTD of NMDA-mediated synaptic currents (Aloisi et al., 2017). Other malfunctions of glutamatergic synapses in *Fmr1* KO mouse brain include a reduced coupling of mGluRs to Homer proteins (Giuffrida et al., 2005), a reduced NMDA/AMPA ratio (Yun and Trommer, 2011; Gocel and Larson, 2012; Aloisi et al., 2017), and altered NMDA-dependent plasticity (Uzunova et al., 2014; Bostrom et al., 2015). An increased expression of Ca^2+^-permeable AMPA receptors was recently found in human neural precursors derived from FXS patients (Achuta et al., 2018). Inhibitory synapses are also affected in the brain of FXS animal models, with a deficit of GABAergic inhibition (Martin et al., 2014; Braat and Kooy, 2015) and abnormal functioning of GABA_A_ receptors (He et al., 2014).

At a cellular level, FMRP absence is associated with dysregulation of many signaling pathways, among which upregulation of PI3K/Akt/mTOR pathway (Sharma et al., 2010; Huber et al., 2015), overactivation of GSK3 (Min et al., 2009), and altered MAPK/ERK signaling (Kim et al., 2008; Osterweil et al., 2010). The large amount of data now available on the molecular basis of FXS provides several cues for a possible therapy of FXS, currently under investigation (Santoro et al., 2012; Sethna et al., 2014; Gross et al., 2015; Castagnola et al., 2017). Each proposed strategy might be useful in subsets of FXS patients, owing to a large individual heterogeneity with respect to the type and severity of symptoms (Jacquemont et al., 2014).

Interestingly, early observations on FXS patients and latest findings on FXS animal models have pointed out a downregulation of the cyclic adenosine monophosphate (cAMP) pathway, originating a “cAMP theory” of FXS (Kelley et al., 2008). A recent study shows that the mRNA coding for phosphodiesterase 2A (PDE2A), a cAMP-degrading enzyme, is among the most prominent targets of FMRP (Maurin et al., 2018a). In the brain of *Fmr1* KO mice, PDE2A is overexpressed and overactive, causing reduced cAMP formation and dysregulation of cAMP downstream signaling (Maurin et al., 2018b). In line with this, synaptic plasticity, learning, and behavior in *Fmr1* KO mice are rescued by agonists of serotonin 5-HT_7_ receptors, positively coupled to adenylate cyclase (Costa et al., 2012, 2015, 2018; Ciranna and Catania, 2014), by PDE4 inhibitors (Choi et al., 2015, 2016), and by a selective PDE2A inhibitor (Maurin et al., 2018b). Of note, inhibition of PDE2A also corrected abnormal dendritic spine morphology of cortical neurons from *Fmr1* KO mice (Maurin et al., 2018b). All these data confirm a deficit in cAMP-mediated signaling in *Fmr1* KO neurons and demonstrate that pharmacological manipulations increasing cAMP levels can rescue synaptic morphology and function, learning, and behavior in animal models of FXS.

Pituitary adenylate cyclase activating polypeptide is a potent stimulator of adenylate cyclase activity (Miyata et al., 1989; Vaudry et al., 2009). Consistent with the cAMP hypothesis of FXS, we found that PACAP reversed mGluR-LTD in the CA3-CA1 hippocampal synapse in wild-type mice and reduced exaggerated mGluR-LTD in *Fmr1* KO, thus correcting a synaptic defect typically observed in FXS mouse models (Costa et al., 2018). This result offers novel suggestions for a possible therapy of FXS, for which no specific cure is presently available. In future studies, it would be interesting to test if PACAP can correct other abnormal features in *Fmr1* KO neurons (dendrite development, synapse formation and function, ion channel expression, membrane excitability, and intracellular signaling) and rescue learning and behavior when administered *in vivo* to *Fmr1* KO mice.

Dysregulation of cyclic nucleotide pathways was found at different levels (synthesis, functioning, and/or degradation by PDE) in aging and age-related cognitive decline (Kelly, 2018). Accordingly, inhibition of PDE activity improved memory in animal models of AD (Gulisano et al., 2018) and HD (Saavedra et al., 2013) and seems to prevent memory loss in elderly humans and in AD patients (Prickaerts et al., 2017). As indicated above, in FXS, the cAMP pathway is also dysregulated, and increasing cAMP rescues several phenotypes. Therefore, altered cAMP signaling might be a common feature in cognitive deficits of very different origin, in which administration of PACAP might be beneficial.

## Systemic Administration Routes for Effective Brain Delivery of Pituitary Adenylate Cyclase-Activating Polypeptide

Pituitary adenylate cyclase activating polypeptide-38 is actively transported into the brain across the blood–brain barrier (BBB) by a saturable carrier, whereas PACAP-27 passes the BBB by transmembrane diffusion (Banks et al., 1993). After intravenous administration, the amount of PACAP-38 brain uptake was high enough to exert neuroprotective effects (Banks et al., 1996). The rate of PACAP-38 transport varies largely in different brain areas, with maximal uptake in the hypothalamus and in the hippocampus (Nonaka et al., 2002), and can be altered in pathological conditions (Banks et al., 1998; Nonaka et al., 2002; Rhea et al., 2018).

On the other side, PACAP-38 and PACAP-27 are transported out of the brain by a common efflux mechanism, reducing their brain levels (Banks et al., 1993). It is noteworthy that antisense inhibition of peptide transport system-6 (PTS-6) improved brain uptake and neuroprotective effects of PACAP-27 in murine models of AD and stroke (Dogrukol-Ak et al., 2009), suggesting that inhibition of PTS-6 efflux component might become a therapeutic strategy to enhance central effects of PACAP.

Some issues were raised about intravenous administration of PACAP to humans: PACAP-38 induced headache in healthy and migraine-suffering subjects (Schytz et al., 2009). Headache was also reported after intravenous infusion of PACAP-27 to healthy subjects (Ghanizada et al., 2019a) and migraine patients (Ghanizada et al., 2019b).

Other issues against intravenous administration of PACAP concern metabolic stability and selectivity. In fact, PACAP-38 showed a very short half-life (<5 min) in human plasma *in vitro*, being converted by the blood enzyme dipeptidyl peptidase IV into shorter peptides that behave as PACAP receptor antagonists, whereas PACAP-27 was relatively stable (Bourgault et al., 2008).

Besides, parenteral administration of PACAP can induce undesired peripheral actions, among which are cardiovascular (Runcie et al., 1995; Farnham et al., 2012) and hormonal effects (Tsutsumi et al., 2002). To overcome these limitations, new synthetic agonists of PACAP receptors have been developed with improved metabolic stability, higher brain uptake, selectivity for PAC1 receptors (the predominant PACAP receptors in the CNS), and reduced side effects (Bourgault et al., 2008; Dejda et al., 2011; Doan et al., 2011; Lamine et al., 2016).

Another strategy exploits conjugation of PACAP with a TAT peptide, improving passage through the BBB (Yu et al., 2012a, b). Carrier vesicles can also be used to protect peptides from blood-degrading enzymes (Dufes et al., 2004).

Administration routes for brain delivery of PACAP are illustrated in details in a recent review (Reglodi et al., 2018a). A promising non-invasive and easy route is intranasal application, by which PACAP reaches the brain fast and effectively in rodents, exerting neuroprotective effects in mouse models of AD (Rat et al., 2011; Nonaka et al., 2012) and HD (Cabezas-Llobet et al., 2018). Intranasal administration of PACAP was also tested on human volunteers, showing good safety and tolerability (Doberer et al., 2007; Reglodi et al., 2018a) with only mild local adverse reactions (Kinhult et al., 2003). Interestingly, headache was not reported after intranasal application of PACAP-38 (Doberer et al., 2007), suggesting this route as a valuable alternative to intravenous administration.

## Concluding Remarks

Pituitary adenylate cyclase activating polypeptide plays an important role in learning and has a therapeutic potential in cognition deficits associated with disruption of cyclic nucleotide signaling. We suggest that the effects of PACAP might also be studied for a possible therapy of FXS, in which a deficit in cAMP formation and downstream signaling were evidenced. For future translational applications, it would be interesting to test *in vivo* effects of PACAP on learning and behavioral deficits in FXS animal models.

Pituitary adenylate cyclase activating polypeptide is brain permeant and has already proved to be safe on healthy humans and thus might be tested in clinical trials. To improve PACAP brain uptake, it would be worth focusing on new selective PAC1 receptor agonists with enhanced metabolic stability, on delivery carriers, and/or on suitable administration routes.

## Author Contributions

LCi designed and directed the research projects and wrote the manuscript. LCo performed the experiments and data analysis, and contributed to the manuscript preparation.

## Conflict of Interest

The authors declare that the research was conducted in the absence of any commercial or financial relationships that could be construed as a potential conflict of interest.
